# Association of *OPRM1 rs1799971, HTR1B rs6296* and *COMT rs4680* polymorphisms with clinical phenotype among women with fibromyalgia

**DOI:** 10.1038/s41598-024-62240-7

**Published:** 2024-05-17

**Authors:** César Fernández-de-las-Peñas, Silvia Ambite-Quesada, Luis M. Fernández-Méndez, Carmen Jiménez-Antona, Cristina Gómez-Calero, Ricardo Pocinho, Juan Antonio Valera-Calero, Margarita Cigarán-Méndez, Lars Arendt-Nielsen

**Affiliations:** 1 Department of Physical Therapy, Occupational Therapy, Physical Medicine and Rehabilitation, Universidad Rey Juan Carlos, Av. de Atenas S/N, 28922 Alcorcón, Madrid, Spain; 2 Center for Neuroplasticity and Pain (CNAP), Department of Health Science and Technology, Faculty of Medicine, SMI, Aalborg University, Aalborg, Denmark; 3 CICS.NOVA. Ipleiria, Instituto Politécnico de Leiria, Leiria, Portugal; 4 Department of Radiology, Rehabilitation and Physiotherapy, Faculty of Nursery, Physiotherapy and Podiatry, Complutense University of Madrid, Madrid, Spain; 5 Grupo InPhysio, Instituto de Investigación Sanitaria del Hospital Clínico San Carlos (IdISSC), Madrid, Spain; 6 Department of Psychology, Universidad Rey Juan Carlos, Madrid, Spain; 7 Department of Gastroenterology & Hepatology, Mech-Sense, Clinical Institute, Aalborg University Hospital, Aalborg, Denmark; 8 Steno Diabetes Center North Denmark, Clinical Institute, Aalborg University Hospital, Aalborg, Denmark

**Keywords:** Single nucleotide polymorphism, Fibromyalgia, Pain genes, Rheumatic diseases, Biomarkers

## Abstract

To investigate the association between three selected pain polymorphisms and clinical, functional, sensory-related, psychophysical, psychological or cognitive variables in a sample of women with fibromyalgia (FMS). One hundred twenty-three (n = 123) women with FMS completed demographic (age, height, weight), clinical (years with pain, intensity of pain at rest and during daily living activities), functional (quality of life, physical function), sensory-related (sensitization-associated and neuropathic-associated symptoms), psychophysical (pressure pain thresholds), psychological (sleep quality, depressive and anxiety level) and cognitive (pain catastrophizing, kinesiophobia) variables. Those three genotypes of the *OPRM1 rs1799971, HTR1B rs6296* and *COMT rs4680* single nucleotide polymorphisms were obtained by polymerase chain reactions from no-stimulated whole saliva collection. No significant differences in demographic, clinical, functional, sensory-related, psychophysical, psychological and cognitive variables according to *OPRM1 rs1799971, HTR1B rs6296* or *COMT rs4680* genotype were identified in our sample of women with FMS. A multilevel analysis did not either reveal any significant gene-to-gene interaction between *OPRM1 rs1799971 x HTR1B rs6296*, *OPRM1 rs1799971 x COMT rs4680* and *HTR1B rs6296 x COMT rs4680* for any of the investigated outcomes. This study revealed that three single nucleotide polymorphisms, *OPRM1 rs1799971, HTR1B rs6296* or *COMT rs4680,* mostly associated with chronic pain were not involved in phenotyping features of FMS. Potential gene-to-gene interaction and their association with clinical phenotype in women with FMS should be further investigated in future studies including large sample sizes.

## Introduction

Fibromyalgia syndrome (FMS) is a complex pain condition affecting up to 6% of the worldwide population^1^. Although the clinical presentation is well described in the literature^2,3^, its etiology is not completely understood. Patients with FMS generally report widespread pain, fatigue, sleep disorders, emotional problems, muscular weakness, cognitive impairments, exacerbated pain responses to painful and non-painful stimuli (defined as hypersensitivity or allodynia, respectively), and decreased physical capacity^4^. It appears the pathogenesis of FMS is complex and several aspects including altered nociceptive processing^5^, distress (e.g., catastrophism, pain hypervigilance, depression, anxiety, kinesiophobia)^6^ and neuropathic pain features^7^ interact at the same time^8^.

Another factor which may be involved in FMS is the presence of one or more genotypes in different single nucleotide polymorphisms^9^. In fact, more than 100 genes have potentially been involved in pain sensitivity and pain-related analgesia^10^. Among potential genes involved in chronic pain, the µ-opioid receptor (*OPRM1*) *rs1799971*, the 5-hydroxytryptamine receptor 1B (*HTR1B*) *rs6296* and the catechol-O-methyltransferase (*COMT*) *rs4680* polymorphisms have been proposed to be associated with FMS^11,12^.

The polymorphism *OPRM1 rs1799971* results in a change of amino acid at position 40, in the first exon of the µ-opioid receptor protein from asparagine (Asn) to be replaced by aspartate acid (Asp). The presence of Asn or Asp allele determines the affinity for altered protein for β-endorphin, which represents a potential mechanism in which this polymorphism may alter pain sensitivity^13^. Ellerbrock et al. did not observe differences on pain sensitivity depending on the genotype of the *OPRM1 rs1799971* polymorphism in people with FMS, although differences in cognitive brain areas were seen^14^. Solak et al.^15^ also did not observe an association between *OPRM1 rs1799971* polymorphism and FMS in a Turkish population. On the contrary, Tour et al. identified that the presence of the Asp allele at the *OPRM1 rs1799971* polymorphism was associated with decreased conditioned pain modulation^16^ and with exercise-induced hypoalgesia in an antagonist effect with the *rs25531* polymorphism of the serotonin transporter (5-HTTLPR) gene in individuals with FMS^17^.

The HTR1B gene codifies the serotonergic receptor (5-HT1B) which is involved in physiological processes such as pain and sleep^18^. The polymorphism *rs6296* results in a synonymous mutation causing a reduction of HTR1B mRNA^18^. Genotypes of the polymorphism *rs6296* has been associated with a modulatory effect on exercise-induced hypoalgesia when combined with *OPRM1 rs1799971* polymorphism in FMS^17^. No previous study has examined if particular genotypes of the *HTR1B rs6296* polymorphism are associated with a higher risk of suffering FMS.

The *Val158Met rs4680* polymorphism results in a substitution of valine (Val) to methionine (Met) in the product of the COMT gene at codon 158^19^ and is probably the genetic factor most frequently investigated in chronic pain conditions. This change causes the production of an enzyme with a lower thermostability (Met/Met genotype) resulting in decreased enzyme activity under physiological conditions^19^. Overall, it has been assumed that people with a Met/Met genotype tend to exhibit higher pain sensitivity than those with the Val/Val genotype^19^; however, it has been recently observed a high heterogeneity in this association supporting that the impact of the *Val158Met rs4680* genotype on pain sensitivity is present when descending pain pathways are sufficiently impaired^20^. In fact, evidence about the association between the *Val158Met rs4680* polymorphism and FMS is also conflicting since Lee et al.^21^ observed higher risk of FMS in carriers of the Met allele of the *Val158Met rs4680* polymorphism whereas Zhang et al.^22^ did not find such association. Discrepancies in the association between the *Val158Met rs4680* polymorphism and FMS continue in more recent studies^23,24^. The lack of a role of this gene on the risk of suffering from FMS does not exclude a potential influence on the clinical course and the severity of the disease. In fact, some studies have investigated the role of the *Val158Met rs4680* polymorphism on related-disability^25^, pain sensitivity^26,27^ or mood disorders^28^ in FMS.

Collectively, these studies have enriched the understanding of FMS and endorse the genetic hypothesis as a contributor to the disease's development and severity, rather than to a higher risk of suffering from FMS^25–28^. Accordingly, given the multifactorial nature of FMS, additional research is warranted to explore the role of diverse genes that may impact disease chronicity and clinical features. Thus, this study aimed to investigate the association of *OPRM1 rs1799971*, *HTR1B rs6296* and *Val/Met COMT rs4680* polymorphisms with clinical, functional, sensory-related, psychophysical, psychological, and cognitive variables in a sample of women with FMS.

## Methods

### Participants

A consecutive sample of women with a diagnosis of FMS participated in this observational cross-sectional study. All participants were recruited from two associations of patients with FMS (AFINSYFACRO and FIBROPARLA Fibromyalgia Associations), located in Madrid (Spain). To be eligible, patients had to present with a medical diagnosis of FMS according to Wolfe et al. modified criteria^29^ and had to be able to read and sign a written informed consent prior to their inclusion in the study. Exclusion criteria included previous whiplash injury, presence of comorbid neuropathic pain (e.g., radiculopathy or myelopathy), had received previous major surgery, other underlying medical condition (e.g., rheumatoid arthritis) or take medication affecting muscle tone or pain perception. We permitted the symptomatic use of non-steroidal anti-inflammatory drugs at the time of the study. The study was approved by the Local Ethics Committee of Universidad Rey Juan Carlos (URJC 08-30-2020) and, in order to enhance the quality of the report, the STROBE guidelines were followed^30^.

### Patient-reported outcome measures (PROMs)

Different PROMs including clinical, functional, sensory-related, cognitive, and psychological variables were collected in a structured questionnaire created for this study.

#### Clinical variables

The mean intensity of pain at rest, the worst level of pain at rest, and intensity of pain experienced during daily living activities were assessed in a 10-point (0: no pain, 10: maximum pain) Numerical Pain Rate Scale (NPRS)^31^.

#### Functional variables

The validated version of the Fibromyalgia Impact Questionnaire (FIQ, score: 0–100 points) in Spanish language, a specific-disease PROM for FMS, was used to assess pain-related disability^32^. The FIQ consists of 10 items (from 0 to 10 points) assessing the function on daily tasks, the number of days feeling good during the previous 7 days, pain interference at work, intensity of pain, fatigue, night resting, presence of stiffness, anxiety and depressive levels. Higher scores represent greater related-disability and pain severity^32^.

The paper-based five-level version of EQ-5D-5L questionnaire, a generic PROM consisting of five dimensions evaluated from 1 (no problem) to 5 (severe problems), was used to determine health-related quality of life^33^. Responses from the EQ-5D-5L are converted into a single index number between 0 (health state judged to be equivalent to death) and 1 (optimal health status) by applying crosswalk index values for the Spanish population^33^.

#### Sensory-related variables

The presence of sensitization-associated symptoms was assessed with the Spanish version of the Central Sensitization Inventory (CSI)^34^. This PROM uses 5-points Likert scale responses to identify the presence of sensitization-related symptoms. The cut-off for determining the presence of altered nociceptive pain processing has been set at 40 points out of total score of 100 points^35^.

The presence of neuropathic-associated pain symptomatology was assessed with the Spanish version of the Self-Report Leeds Assessment of Neuropathic Symptoms (S-LANSS) questionnaire^36^. This PROM identifies with acceptable sensitivity, internal consistency and validity whether a patient experiences pain symptom to be considered predominantly or non-predominantly of neuropathic origin^36^. From a total score of 24 points, scores higher than 12 points suggest the presence of neuropathic symptoms^36^.

#### Psychological variables

The Hospital Anxiety and Depression Scale (HADS) was used to evaluate the levels of anxiety (HADS-A, 7-items) and depression (HADS-D, 7-items) in our sample of FMS^37^. A score of 12 points out of 21 in the HADS-A and a score of 10 points out of 21 in the HADS-D suggests the presence of anxiety and depressive levels, respectively^37^.

The quality of sleep was assessed with the Pittsburgh Sleep Quality Index (PSQI)^38^. This PROM uses 4-point Likert-type scale responses evaluating different aspects of sleep, where 8 points out of a total score of 21 suggest poor sleep quality^38^.

#### Cognitive variables

The Spanish version of the Pain Catastrophizing Scale (PCS), a PROM consisting of 13 items answered in a 5-point Likert scale (total scores range from 0 to 52), was used to assess catastrophizing responses (e.g., rumination, magnification, and despair aspects)^39^.

Fear of movement perceived by the patient was assessed with the Spanish version of the 11-items short-form of the Tampa Scale for Kinesiophobia (TSK-11)^40^. This PROM includes 11 items assessing the attitude of the patient against pain answered with a 4-point Likert scale (1: “complete disagreement” to 4: “complete agreement”), leading to a score ranging from 0 to 44 points.

### Pressure pain thresholds

Pressure pain thresholds (PPTs) were bilaterally assessed at the upper trapezius, second metacarpal and tibialis anterior muscle with an electronic algometer (Somedic AB©, Farsta, Sweden) to identify the presence of widespread pressure pain sensitivity as a clinical manifestation of altered nociceptive pain processing. Pressure was applied at a rate of approximately 30 kPa/s on each point, three times on each location with a 30-s resting period between measurements. For analyses, the mean score of the three trials on each point was calculated. This testing procedure has shown good reliability (ICC ≥ 0.88) in patients with FMS^41^.

### DNA collection and genotyping

Unstimulated whole saliva samples were always collected during the morning into collection tubes according to standardized procedures. Participants were asked not to eat, drink or chew gum for 1 h before sample collection. The saliva samples were collected, stored and centrifuged at 3000 rpm for 15 min no later than 48 h after collection to obtain the cell sediment and stored at -20º C until the analysis. This process is the same as used in previous studies^28,42^.

Genomic DNA was extracted from 500 ml of saliva using a MagMAX™ DNA Multi-Sample Ultra 2.0 Kit (Thermo Fisher Scientific Inc, Hemel Hempstead, Hertfordshire, UK) according to the manufacturer’s protocol. We automatically extracted DNA using the King Fisher Flex purification robot (Thermo Fisher). The resulting DNA was assessed for purity and concentration using Quant-iT™ PicoGreen™ dsDNA reagent" (Thermo Fisher). The resulting DNA was diluted to 5 ng/μl, using 1 × Tris‐EDTA (TE) buffer (Sigma‐Aldrich, Dorset, UK). The qPCR reaction mixtures of 10ul contained a total of 10 ng gDNA as a PCR template, 1 × TaqMan Gene Expression PCR Master Mix and 0.6 × Genotyping TaqMan-probe assay.

For genotyping single nucleotide polymorphisms (SNP) with Real-Time PCR reaction TaqMan® Assay ID C_25746809_50 Predesigned SNP Genotyping Assays was used (Thermo Fisher Scientific Inc, Hemel Hempstead, Hertfordshire, UK). TaqMan® SNP Genotyping Assays use TaqMan® 5’‐nuclease chemistry for amplifying and detecting specific alleles of each SNP in purified genomic DNA samples. Each assay allows the genotyping of individuals for a single nucleotide polymorphism. Real-time PCR plates were run in the Quantstudio 12 K Flex System (Thermo Fisher) of Genomics Unit (Madrid Science Park Foundation, Spain) under standard running conditions (95° for 10 min and 40 two-step cycles consisting of 95 °C for 15 s and 60 °C for 1 min) and analyzed with Genotyping App of Thermo Fisher Cloud. Identification of each of the three possible variants of each single nucleotide polymorphism was conducted by using specific fluorescent dyes.

The possible variants of the *OPRM1 rs1799971* polymorphism lead to the following genotypes: *Asn/Asn (A/A), Asn/Asp (A/G) or Asp/Asp (G/G).* The results were derived from a A → G substitution at the following sequence:

GGTCAACTTGTCCCACTTAGATGGC [A/G] ACCTGTCCGACCCATGCGGTCCGAA

The variants of the *HTR1B rs6296* polymorphism lead to the following genotypes: *C/C*, *C/G* or *G/G*. The results were derived from a C → G substitution at the sequence:

CGGAGACTCGCACTTTGACTTGGTT [C/G] ACATACACAGGAGATCCGGATTCGC

The possible variants of the *COMT rs4680* polymorphism lead to the following three genotypes: *Val/Val (G/G), Val/Met (G/A), Met/Met (A/A).* The results were derived from A → G substitution at the following sequence:

CCAGCGGATGGTGGATTTCGCTGGC [G/A] TGAAGGACAAGGTGTGCATGCCTGA.

### Statistical analysis

Statistical analyses were performed using SPSS Statistics, version 25.0. Data were expressed as mean ± standard deviation (SD). The chi-squared (χ2) test was used to assess potential deviations from Hardy–Weinberg equilibrium. The Shapiro–Wilk test revealed that data exhibited normal distribution, hence, parametric tests were used. First, separate analyses of variance (ANOVA) were used to assess differences in clinical, functional, sensory-related, cognitive, and psychological variables according to the genotype of each polymorphism (*OPRM1 rs1799971, HTR1B rs6296*, *COMT rs4680*). Second, multilevel analysis of variance (MANOVA) including all polymorphisms (*OPRM1 rs1799971, HTR1B rs6296*, *COMT rs4680*) was also conducted to identify the possible interaction between all combinations of genotypes in the outcomes. Due to the inclusion of several variables, we applied correction by multiple testing considering a Bonferroni-corrected alpha of 0.0125 (4 time points).

### Ethical approval

The study was conducted according to the guidelines of the Declaration of Helsinki and approved by the Clinical Ethics Committee of Universidad Rey Juan Carlos (URJC 08–30-2020). Written informed consent was obtained from all subjects involved in the study.

## Results

From 140 Caucasian women with a diagnosis of FMS screened for eligible criteria, 14 (10%) were excluded due to previous major surgery (n = 8), previous whiplash (n = 4), and pregnancy (n = 2), leading to a final sample of 126 patients (mean age: 52 ± 9.9 years old). Three saliva samples were undetermined during genotyping analysis for the investigated SNPs and, accordingly, were excluded from the analysis providing a sample of 123 women with FMS with the complete data (Table 1).

**Table 1:** Clinical, functional, sensory-related, cognitive, psychological, and psychophysical data of the sample.

	Mean	SD	Min	Max
*Demographic features*				
Age (years)	52.0	9.9	42.0	65.0
Height (cm)	161.00	6.5	155.0	170.0
Weight (kg)	71.00	14.4	57.0	95.0
*Clinical features*				
Years with diagnosis	12.9	5.6	8.0	17.0
Mean pain (NPRS, 0–10)	6.4	1.7	3.0	9.0
Worst pain (NPRS, 0–10)	7.3	2.2	5.0	10.0
Pain with activity (NPRS, 0–10)	8.1	1.9	2.0	10.0
*Functional variables*				
FIQ (0–100)	64.7	13.4	25.0	92.5
Quality of life (EQ-5DL, 0–1)	0.4	0.25	0.1	0.9
*Sensory-related variables*				
CSI Score (0–100)	70.5	11.9	36.0	99.0
S-LANSS (0–24)	17.5	5.2	5.0	28.0
*Cognitive variables*				
Catastrophizing (PCS, 0–52)	22.0	12.5	9.5	37.0
Kinesiophobia (TSK-11)	24.8	7.5	11.0	43.0
*Psychological variables*				
HADS-A (0–21)	11.5	4.0	3.0	20.0
HADS-D (0–21)	9.9	4.1	2.0	18.0
Sleep (PSQI, 0–21)	13.5	4.0	5.0	20.0
*Psychophysical features—pressure pain thresholds*				
PPT upper trapezius (kPa)	130.1	48.4	50.45	273.8
PPT second metacarpal (kPa)	120.2	48.2	35.5	254.0
PPT tibialis anterior (kPa)	189.2	89.0	55.5	245.8

NPRS: Numerical Pain Rate Scale; FIQ: Fibromyalgia Impact Questionnaire; CSI: Central Sensitization Inventory; S-LANSS: self-reported version of the Leeds Assessment of Neuropathic Symptoms and Signs; PCS: Pain Catastrophizing Scale; TSK-11: Tampa Scale for Kinesiophobia; HADS: Hospital Anxiety and Depression Scale (A: Anxiety; D: Depression); PSQI: Pittsburgh Sleeping Quality Index; PPT: Pressure Pain Thresholds.

The *Asn/As*n genotype of the *OPRM1 rs1799971* polymorphism was the most prevalent (63.4%), followed by the *Asn/Asp* (31.7%) and the *Asp/Asp* (4.9%) genotypes (Table 2). Thus, the *Asn* allele was present in 79.2% (n = 195/246) whereas the *Asp* allele was present in the remaining 20.8% (n = 51/246) of the sample. This genotype/allele distribution of the *OPRM1 rs1799971* polymorphism did not deviate from that expected based on the Hardy–Weinberg equilibrium (chi square = 0.153).

**Table 2:** Differences in Clinical, Functional, Sensory-Related, Cognitive and Psychological variables as well as Pressure Pain Sensitivity in women with Fibromyalgia Syndrome Depending on the µ-Opioid Receptor Gene (OPRM1) Polymorphism (*rs1799971).*

	Asn/Asn (n = 78)	Asn/Asp (n = 39)	Asp/Asp (n = 6)	P value
*Demographic features*				
Age (years)	53.0 ± 10.0	51.0 ± 12.0	50.0 ± 15.5	0.466
Height (m)	1.61 ± 0.1	1.62 ± 0.1	1.60 ± 0.1	0.539
Weight (kg)	70.5 ± 14.0	73.5 ± 20.0	70.0 ± 12.0	0.347
*Clinical features*				
Years with pain	9.8 ± 4.2	10.0 ± 5.5	11.0 ± 7.6	0.444
Mean Pain intensity (NPRS, 0–10)	6.3 ± 1.7	6.6 ± 1.7	6.4 ± 1.4	0.525
Worst Pain Intensity (NPRS, 0–10)	7.4 ± 2.5	8.0 ± 1.8	7.8 ± 2.1	0.158
Pain during Activities (NPRS, 0–10)	7.8 ± 1.9	8.5 ± 1.6	8.2 ± 2.1	0.077
*Functional variables*				
FIQ (0–100)	63.1 ± 12.8	67.5 ± 12.3	67.3 ± 14.4	0.167
EQ-5D-5L (0–1)	0.4 ± 0.25	0.4 ± 0.25	0.45 ± 0.3	0.415
*Sensory-related variables*				
CSI (0–100)	70.3 ± 11.3	70.2 ± 17.3	72.7 ± 11.1	0.686
S-LANSS (0–24)	17.4 ± 5.1	18.1 ± 5.9	17.0 ± 6.0	0.644
*Cognitive variables*				
PCS (0–52)	22.2 ± 12.5	23.8 ± 10.9	23.8 ± 16.0	0.791
TSK-11 (0–44)	25.0 ± 7.3	25.5 ± 7.8	24.6 ± 7.2	0.945
*Psychological variables*				
HADS-A (0–21)	11.4 ± 3.8	11.1 ± 3.7	11.6 ± 3.2	0.888
HADS-D (0–21)	10.0 ± 4.0	9.6 ± 4.1	11.4 ± 4.2	0.609
PSQI (0–21)	14.2 ± 3.9	13.8 ± 3.7	14.2 ± 4.0	0.612
*Pressure pain thresholds (PPT)*				
PPT trapezius muscle (kPa)	123.0 ± 62.0	134.0 ± 60.0	100.0 ± 30.5	0.344
PPT second metacarpal (kPa)	121.0 ± 59.0	118.5 ± 58.0	133.5 ± 67.5	0.654
PPT tibialis anterior (kPa)	193.0 ± 122.0	172.5 ± 81.5	191.0 ± 79.0	0.485

NPRS: Numerical Pain Rate Scale; FIQ: Fibromyalgia Impact Questionnaire; CSI: Central Sensitization Inventory; S-LANSS: self-reported version of the Leeds Assessment of Neuropathic Symptoms and Signs; PCS: Pain Catastrophizing Scale; TSK-11: Tampa Scale for Kinesiophobia; HADS: Hospital Anxiety and Depression Scale (A: Anxiety; D: Depression); PSQI: Pittsburgh Sleeping Quality Index; PPT: Pressure Pain Thresholds.

The ANOVA did not reveal any significant difference in demographic (age, height, weight), clinical (pain history, mean pain, worst pain, pain on daily activities) functional (FIQ, EQ-5D-5L), sensory-related (CSI, S-LANSS), PPTs (upper trapezius, second metacarpal, tibialis anterior), psychological (HADS-A, HADS-D, PSQI), and cognitive (PCS, TSK-11) variables depending on the *OPRM1 rs1799971* genotype (Table 2).

The *C/C* genotype of the *HTR1B rs6296* polymorphism was present in 51.2% (n = 63) of the sample, whereas the *C/G* and *G/G* genotypes were present in 41.4% (n = 51) and 7.4% (n = 9) of the sample (Table 3). Thus, the *C* allele was present in 71.9% (n = 177/246) whereas the *G* allele was present in the remaining 28.1% (n = 69/246) of the sample. This genotype/allele distribution of the *HTR1B rs6296* polymorphism did not deviate from that expected based on the Hardy–Weinberg equilibrium (chi square = 0.091).

**Table 3:** Differences in Clinical, Functional, Sensory-Related, Cognitive and Psychological variables as well as Pressure Pain Sensitivity in women with Fibromyalgia Syndrome Depending on the 5-hydroxytryptamine receptor 1B gene (HTR1B) Val287 Polymorphism (*rs6296*).

	C/C (n = 63)	C/G (n = 51)	G/G (n = 9)	P value
*Demographic features*				
Age (years)	51.5 ± 10.5	52.5 ± 10.0	54.5 ± 14.0	0.270
Height (m)	1.61 ± 0.1	1.60 ± 0.1	1.63 ± 0.1	0.581
Weight (kg)	73.0 ± 16.0	71.0 ± 18.0	70.0 ± 14.0	0.563
*Clinical features*				
Years with pain	9.0 ± 3.1	11.5 ± 6.5	11.0 ± 3.6	0710
Mean Pain intensity (NPRS, 0–10)	6.3 ± 1.7	6.5 ± 1.6	6.6 ± 1.8	0.544
Worst Pain Intensity (NPRS, 0–10)	7.5 ± 2.0	7.0 ± 2.4	7.5 ± 1.5	0.443
Pain during Activities (NPRS, 0–10)	7.8 ± 2.1	8.4 ± 1.5	8.3 ± 2.1	0.253
*Functional variables*				
FIQ (0–100)	62.7 ± 11.8	66.5 ± 13.1	66.7 ± 13.7	0.240
EQ-5D-5L (0–1)	0.4 ± 0.25	0.45 ± 0.2	0.5 ± 0.3	0.583
*Sensory-related variables*				
CSI (0–100)	69.5 ± 11.2	71.2 ± 12.0	73.2 ± 12.3	0.472
S-LANSS (0–24)	17.4 ± 5.1	18.1 ± 5.9	17.0 ± 6.0	0.424
*Cognitive variables*				
PCS (0–52)	23.4 ± 12.3	24.5 ± 11.4	25.0 ± 12.9	0.305
TSK-11 (0–44)	24.0 ± 7.0	26.0 ± 7.8	25.5 ± 8.1	0.358
*Psychological variables*				
HADS-A (0–21)	11.2 ± 3.4	11.3 ± 4.0	12.5 ± 4.1	0.634
HADS-D (0–21)	10.0 ± 3.8	10.0 ± 4.1	10.0 ± 5.2	0.958
PSQI (0–21)	14.4 ± 3.8	13.0 ± 4.0	14.0 ± 4.5	0.183
*Pressure pain thresholds (PPT)*				
PPT trapezius muscle (kPa)	125.5 ± 60.5	127.0 ± 63.0	130.5 ± 47.5	0.972
PPT second metacarpal (kPa)	120.0 ± 57.5	125.5 ± 63.5	120.0 ± 48.5	0.707
PPT tibialis anterior (kPa)	188.5 ± 108.0	192.5 ± 115.5	182.5 ± 74.5	0.875

NPRS: Numerical Pain Rate Scale; FIQ: Fibromyalgia Impact Questionnaire; CSI: Central Sensitization Inventory; S-LANSS: self-reported version of the Leeds Assessment of Neuropathic Symptoms and Signs; PCS: Pain Catastrophizing Scale; TSK-11: Tampa Scale for Kinesiophobia; HADS: Hospital Anxiety and Depression Scale (A: Anxiety; D: Depression); PSQI: Pittsburgh Sleeping Quality Index; PPT: Pressure Pain Thresholds.

Again, no significant difference in demographic (age, height, weight), clinical (pain history, mean pain, worst pain, pain on daily activities) functional (FIQ, EQ-5D-5L), sensory-related (CSI, S-LANSS), psychophysical (PPT upper trapezius, PPT second metacarpal, PPT tibialis anterior), psychological (HADS-A, HADS-D, PSQI), and cognitive (PCS, TSK-11) variables were identified depending on the *HTR1B rs6296* genotype (Table 3).

The *Val/Val* genotype of the *COMT rs4680* polymorphism was present in 27.6% (n = 34) of the sample, whereas the *Val/Met* and *Met/Met* genotypes were present in 49.7% (n = 61) and 22.7% (n = 28) of the sample, respectively (Table 3). Thus, the *Val* allele was present in 52.4% (n = 129/246) whereas the *Met* allele was present in the remaining 47.6% (n = 117/246) of the sample. This genotype and allele distribution of the *COMT rs4680* polymorphism deviated from that expected based on the Hardy–Weinberg equilibrium (chi square = 0.042).

The ANOVA did not either reveal any significant difference in demographic (age, height, weight), clinical (pain history, mean pain, worst pain, pain on daily activities) functional (FIQ, EQ-5D-5L), sensory-related (CSI, S-LANSS), PPTs (upper trapezius, second metacarpal, tibialis anterior), psychological (HADS-A, HADS-D, PSQI), and cognitive (PCS, TSK-11) variables depending on the COMT *rs4680* genotype (Table 4).

**Table 4:** Differences in Clinical, Functional, Sensory-Related, Cognitive and Psychological variables as well as Pressure Pain Sensitivity in women with Fibromyalgia Syndrome Depending on the Catechol-O-Methyltransferase Gene (COMT) Val158Met Polymorphism (*rs4680).*

	Val/Val (n = 34)	Val/Met (n = 61)	Met/Met (n = 28)	P value
*Demographic features*				
Age (years)	55.0 ± 9.5	53.0 ± 10.5	54.0 ± 12.0	0342
Height (m)	1.61 ± 0.05	1.62 ± 0.05	1.60 ± 0.05	0.358
Weight (kg)	73.0 ± 19.0	72.0 ± 15.0	70.0 ± 15.5	0.341
*Clinical features*				
Years with pain	9.1 ± 4.8	8.0 ± 3.5	15.0 ± 3.2	0.045
Mean Pain intensity (NPRS, 0–10)	6.1 ± 2.0	6.4 ± 1.4	6.7 ± 1.7	0.175
Worst Pain Intensity (NPRS, 0–10)	7.1 ± 2.1	7.4 ± 2.3	7.3 ± 2.1	0.734
Pain during Activities (NPRS, 0–10)	8.2 ± 2.1	8.2 ± 1.5	7.9 ± 2.2	0.499
*Functional variables*				
FIQ (0–100)	60.2 ± 13.5	64.6 ± 10.5	67.3 ± 12.7	0.033
EQ-5D-5L (0–1)	0.45 ± 0.25	0.35 ± 0.25	0.45 ± 0.25	0.317
*Sensory-related variables*				
CSI (0–100)	67.5 ± 11.9	69.3 ± 11.5	73.1 ± 10.9	0.036
S-LANSS (0–24)	18.4 ± 5.1	17.6 ± 5.1	16.0 ± 5.6	0.198
*Cognitive variables*				
PCS (0–52)	21.3 ± 12.5	20.5 ± 11.2	24.6 ± 12.5	0.125
TSK-11 (0–44)	22.8 ± 7.5	25.5 ± 7.1	25.5 ± 7.7	0.255
*Psychological variables*				
HADS-A (0–21)	10.4 ± 3.4	10.6 ± 4.0	12.5 ± 3.5	0.113
HADS-D (0–21)	9.1 ± 3.7	10.4 ± 4.2	10.1 ± 4.2	0.348
PSQI (0–21)	13.0 ± 3.6	13.8 ± 3.8	14.5 ± 4.4	0.330
*Pressure pain thresholds (PPT)*				
PPT trapezius muscle (kPa)	123.0 ± 61.0	123.0 ± 61.0	134.0 ± 60.0	0.719
PPT second metacarpal (kPa)	125.5 ± 63.5	118.5 ± 61.0	119.0 ± 51.5	0.820
PPT tibialis anterior (kPa)	206.5 ± 117.0	180.5 ± 95.5	185.5 ± 88.0	0.458

NPRS: Numerical Pain Rate Scale; FIQ: Fibromyalgia Impact Questionnaire; CSI: Central Sensitization Inventory; S-LANSS: self-reported version of the Leeds Assessment of Neuropathic Symptoms and Signs; PCS: Pain Catastrophizing Scale; TSK-11: Tampa Scale for Kinesiophobia; HADS: Hospital Anxiety and Depression Scale (A: Anxiety; D: Depression); PSQI: Pittsburgh Sleeping Quality Index; PPT: Pressure Pain Thresholds.

Finally, the multivariate analysis did not reveal any statistical significant gene-to-gene interactions between *OPRM1 rs1799971 x HTR1B rs6296*, *OPRM1 rs1799971 x COMT rs4680* and *HTR1B rs6296 x COMT rs4680* for any of the investigated outcomes.

## Discussion

This study examined the role of three polymorphisms, i.e., *OPRM1 rs1799971, HTR1B rs6296* and COMT *rs4680*, commonly related to chronic pain within the clinical phenotype in women with FMS. Current results revealed that the *OPRM1 rs1799971, HTR1B rs6296* and COMT *rs4680* were not overall involved in phenotyping features of FMS related-pain.

### OPRM1 gene and fibromyalgia

Previous research has found that healthy people with the *Asp* allele exhibit lower pressure pain sensitivity compared with those carrying the *Asn* allele associated with the *OPRM1 rs1799971* polymorphism^43^. This change is related to an alteration of the µ-opioid receptor, reducing or eliminating a putative site for N-linked glycosylation, leading to potentially altered interaction with β-endorphins^43^. In the current study, genotypes associated with the *OPRM1 rs1799971* polymorphism did not show association with any of the investigated variables in our sample of women with FMS. Further, a previous study did not either find an association between *OPRM1 rs1799971* polymorphism and a higher risk of developing FMS^15^. Current and previous findings would suggest that *OPRM1 rs1799971* polymorphism genotypes are not related to pain in a specific chronic condition such as FMS. It is possible a potential effect of different or more than one type of opioid receptor, or a gene-to-gene interaction of this polymorphism in the clinical phenotype in women with FMS.

### HTR1B gene and fibromyalgia

Similarly, we also observed that *HTR1B rs6296* polymorphism, involved in the regulation of serotonin levels by inducing a synonymous mutation on the *Val* in position 287^18^, was not either associated with the clinical phenotype in women with FMS when investigated in isolation. Although this polymorphism has been associated with mental problems^44^, we did not either find significant differences in psychological or cognitive variables according to its genotypes. However, we do not know if other mental problems, such as suicide ideation^45^ or the presence of post-traumatic stress disorder (PTSD)^46^, which are commonly seen in FMS, would be associated with these polymorphisms in this population.

### COMT gene and fibromyalgia

The *Val158Met rs4680* is the polymorphism most investigated in FMS; however, its association with a higher risk of suffering this condition is controversial^21–24^. The *Met* allele of this polymorphism results in an increased degradation of neurotransmitters such as dopamine, norepinephrine or epinephrine and, therefore, a reduction of these neurotransmitters at synaptic receptors^19^. We observed that women with FMS carrying the *Met/Met* genotype tend to report longer pain history, lower function and sensitization -associated symptoms than those with the other genotypes; however, differences did not reach the statistical significance. Our results disagree with previous studies showing that patients with FMS and *Met/Met* carriers exhibit higher related-disability^25^ and worse psychological aspects^28^. Similarly, an association with higher pain sensitivity, as a manifestation of central sensitization has been also associated with the *Met/Met* genotype in previous studies^26,27^. Nevertheless, it is important to consider that COMT variation has long been known to be more complex than the single Val/Met polymorphism and studies looking at haplotypes of multiple SNPs are now necessary to account for this complexity.

### Other polymorphisms and fibromyalgia

The fact that the investigated polymorphisms are not related to the pain phenotype or a higher risk of suffering a particular condition, in such a case FMS, does not exclude a genetic component in this pain condition. For instance, a comprehensive genome-wide investigation underscored that immediate family members are more susceptible to FMS, bolstering the assumption of a genetic foundation^47^. Potential genes accountable for pain susceptibility in FMS are the serotonin transporter gene (*SLC64A4*) and the transient receptor potential vanilloid 2 (*TRPV2*) gene^48^. In fact, *SLC64A4*, marked by a single nucleotide polymorphism, has been linked to chronic pain conditions such as temporo-mandibular pain as well as to elevated depression levels, and psychological disorders due to changes in serotonin reuptake^49^. Similarly, *TRPV2* gene, which is expressed in mechano- and thermo-responsive neurons in the dorsal root and trigeminal ganglia, could be associated with the decreased pain thresholds observed in patients with FMS^11^.

It is also possible that other type of genetic analysis, such as DNA methylation, could reveal potential association between different genes and FMS. In fact, *GRM2* methylation has revealed that this gene plays a relevant role in FMS patients since it causes an increase of cerebrospinal fluid glutamate levels^48^. Therefore, future studies investigating the association of these genes with the clinical phenotype in people with FMS are needed.

### Limitations

Finally, it is important to note some potential limitations to the current study. First, the sample only included women with FMS. Hence, extrapolation of these results to men with FMS should not be done. Therefore, investigating sex differences in future studies is needed. In addition, all participants were Caucasian, so we were not able to investigate the role of race or ethnicity in genetics. Lee et al. found that African-Americans were more likely to exhibit a Met/Met genotype than other ethnics, regardless of the diagnosis of FMS^24^. Thus, by excluding individuals with FMS taking analgesic drugs (aside from NSAIDs), we could unintentionally excluded patients with more pain and pain-related comorbidities. Accordingly, our results should be considered according to the features of our sample. Second, the sample size could be considered relatively small, and we did not conduct “a priori” sample size calculation leading to the identification of small samples (n = 6 or n = 9) in some allele homozygote genotypes which could be quite underpowered. Third, we did not include a control group; nevertheless, since we assessed the phenotype of a chronic pain condition, the inclusion of a pain-free control group would have not permitted assessment of these pain-related outcomes. Finally, we only assessed three polymorphisms associated with altered nociceptive processing. Future studies should investigate the influence of different polymorphisms, in addition to other genes in the clinical phenotype of this condition.

## Conclusion

This study examined the role of three polymorphisms, i.e., *OPRM1 rs1799971, HTR1B rs6296* and COMT *rs4680*, commonly related to chronic pain within the clinical phenotype in women with FMS. Current results revealed that the *OPRM1 rs1799971, HTR1B rs6296* and COMT *rs4680* were not overall involved in phenotyping features of FMS related-pain. Future studies including large sample sizes and a greater number of polymorphisms are needed.

## Data Availability

All data derived from this study are presented in the text. Data are available from the corresponding author upon reasonable request.
